# Semen quality and cigarette smoking in a cohort of healthy fertile men

**DOI:** 10.1097/EE9.0000000000000055

**Published:** 2019-06-25

**Authors:** Qiuqin Tang, Feng Pan, Xian Wu, Cody E. Nichols, Xinru Wang, Yankai Xia, Stephanie J. London, Wei Wu

**Affiliations:** a Department of Obstetrics, The Affiliated Obstetrics and Gynecology Hospital of Nanjing Medical University, Nanjing Maternity and Child Health Care Hospital, Nanjing, People’s Republic of China; b Department of Urology, The Affiliated Obstetrics and Gynecology Hospital of Nanjing Medical University, Nanjing Maternity and Child Health Care Hospital, Nanjing, People’s Republic of China; c National Toxicology Program Laboratory, Division of the National Toxicology Program, National Institute of Environmental Health Sciences, Research Triangle Park, Durham, NC; d National Institute of Environmental Health Sciences, National Institutes of Health, Department of Health and Human Services, Research Triangle Park, Durham, NC; e State Key Laboratory of Reproductive Medicine, Department of Toxicology, School of Public Health, Nanjing Medical University, Nanjing, People’s Republic of China; f Key Laboratory of Modern Toxicology of Ministry of Education, Nanjing Medical University, Nanjing, People’s Republic of China.

## Abstract

**Background:**

Numerous health effects of smoking are well-known; associations with semen quality are uncertain. Most previous studies did not adjust for potential confounders and had limited information on age at smoking initiation or smoking cessation.

**Methods:**

We investigated 1,631 healthy fertile men in the Nanjing Medical University Longitudinal Investigation of Fertility and the Environment (NMU-LIFE) study. Relationships were examined using multivariable linear regression controlling for potential covariates.

**Results:**

We found a significant decrease in semen volume (*β* = −0.10, *P* = 0.001) and total sperm count (*β* = −0.42, *P* = 0.037), and significant increase in total motility (*β* = 6.02, *P* = 0.037) and progressive motility (*β* = 5.52, *P* = 0.037) in ever smokers of pack-years ≥10 compared with never smokers. We observed an inverse dose-dependent relation between smoking pack-years and semen volume (*P* < 0.001) and total sperm count (*P* = 0.010) and a positive dose-dependent relation between smoking pack-years and both total motility and progressive motility (*P* = 0.042 and 0.048, respectively). No significant differences in semen quality were detected among ever smokers with different ages at smoking initiation nor in former smokers compared with never smokers.

**Conclusions:**

Cigarette smoking was associated with lower semen volume and total sperm count and higher sperm motility. Smoking cessation might have a restorative effect on semen quality. This finding has important implications for public health research and for understanding the development of abnormal semen quality.

## Abstract 2

Supplemental Digital Content is available in the text.

**What this study adds**

We examined the dose–response association of cigarette smoking and semen quality in a large cohort of healthy fertile young men. Cigarette smoking is associated with lower semen volume and total sperm count and higher sperm motility in fertile men, especially in heavy smokers. The detrimental effects of smoking on semen quality were not seen in men who had stopped smoking. This work has implications for public health research to help shape opinions related to smoking and male fertility and may shed light on how environmental exposures can impact the development of abnormal semen quality.

## Introduction

According to the World Health Organization (WHO), one third of the world’s population over 15 years of age smokes cigarettes.^1^ Cigarette smoking is one of the most important lifestyle-associated risk factors involved in human diseases.^2,3^ Previous studies have shown that smoking is associated with important negative effects on reproductive health both in men and women.^4^ Experimental studies in animal models have indicated that cigarette smoking could be directly or indirectly toxic to spermatogenesis.^5,6^

Semen analysis is the most important diagnostic tool used to assess fertility and includes parameters such as semen volume, sperm concentration, sperm count, sperm motility, and sperm motion.^7^ Multiple studies have suggested that semen quality has declined worldwide;^8–10^ however, the reasons for the decline remain largely unknown. An association between semen quality and smoking has been reported in a number of studies, but the results are inconsistent.^11–15^ Most of the previous studies were limited to relatively small groups of infertile males or males with unknown fertility status. A meta-analysis of studies encompassing 5865 men from 26 countries/regions identified smoking as a risk factor for semen quality in both fertile and infertile groups.^16^ Only seven studies used the most recent WHO criteria (2010) then available. The largest sample size of fertile men in the meta-analysis is just 60. In contrast, a review by Marinelli et al^17^ concluded that smoking has limited effects on semen quality. Therefore, the relation between cigarette smoking and semen quality is still under debate, and large-scale studies of semen quality encompassing the variety of smoking patterns among the general population of fertile men are needed.

To our knowledge, there is no study investigating the association between smoking and semen quality in a large population of fertile men. Additionally, no studies have tested the impact of age at smoking initiation and smoking cessation on semen quality. Of note, it has been recently suggested that the impact of a father who smoked on offspring health may be greatest when the father initiated smoking before puberty.^18^ Additionally, from a public health perspective, if smoking is indeed related to lower semen quality, it is important to know if this association remains in former smokers, but there are no data. Therefore, we investigated the association between various aspects of cigarette smoking history and semen quality among 1631 fertile men from the Nanjing Medical University Longitudinal Investigation of Fertility and the Environment (NMU-LIFE) study.

## Methods

### Study population

All participants were enrolled in the NMU-LIFE study, a larger cohort study of the impact of environmental agents on reproductive health in Nanjing, China. Pregnant women were targeted for recruitment of the family unit by the pregnant woman’s maternity care doctor. Approximately 93% of identified women agreed to participate. Written informed consent was obtained for all interested participants. The NMU-LIFE study population consisted of 2090 fertile men from 2010 to 2016. Excluding those with a history of cryptorchidism, hypospadias, and varicocele, this study includes 1631 participants for whom data on semen quality, detailed information on smoking, and complete covariates were all available. All protocols and informed consent were approved by the Institutional Ethics Committee of Nanjing Medical University.

### Quantification of smoking

Information on smoking status and habits was obtained using a self-administered questionnaire. Participants were classified as current smokers if they were currently smoking and had been smoking for at least 1 year before interview, former smokers if they had quit smoking for at least 6 months and had smoked for at least 1 year, and never smokers were men who never smoked. Participants reported their history of passive smoking exposure as the average number of hours per day: 0, 0–0.5, 0.5–1, and >1. Both former and current smokers were asked to report the usual number of cigarettes smoked per day, for how many years they had smoked that amount, age at smoking initiation, and years after smoking cessation in former smokers. Participants were divided into different groups according to smoking status, smoking intensity, duration, cumulative dose of smoking, and age at smoking initiation. Among former and current smokers, cumulative dose of smoking was stratified into <5, 5–10, and ≥10 pack-years.

### Semen collection and analysis

All semen samples were collected during the second trimester of the participant’s spouse pregnancy. Men were instructed to collect semen samples by masturbation into sterile plastic specimen cups in a semen collection room. Semen specimens were then liquefied at 37°C and immediately analyzed using computer-aided semen analysis (CASA, WLJY 9000, Weili New Century Science and Tech Dev) according to the WHO guidelines (WHO, 2010). Sperm outcomes include semen volume (ml), sperm concentration (×10^6^/ml), total sperm count (×10^6^/ml), total motility (%), progressive motility (%), and sperm motion parameters. Sperm motion measures are curvilinear velocity (VCL), straight-line velocity (VSL), linearity (LIN), average path velocity (VAP), wobble (WOB), straightness (STR), mean angular displacement (MAD), beat cross frequency (BCF), and amplitude of lateral head displacement (ALH).

### Statistical analysis

Unpaired two-sided Student’s *t* tests were used to compare the means of normally distributed continuous parameters. In all other cases, the Mann–Whitney *U* test was used for comparisons between the groups. Box–Cox transformation was used to determine the optimal transformation for each variable of semen quality. Specifically, we found that semen quality parameters required log transformation (log10) (i.e., semen volume), cubic root transformation (i.e., total sperm count), square root transformation (i.e., ALH), or no transformation (i.e., sperm concentration, total motility, progressive motility, VCL, VSL, LIN, VAP, WOB, STR, MAD, and BCF). Linear regression models were used to assess how smoking is related to semen quality. These models were run unadjusted, and with adjustment for potential confounding variables. Confounding variables in the adjusted models included age (continuous), body mass index (BMI) (continuous), ethnicity (Han/other), education attainment (high school and below/college degree and above), alcohol drinking status (never drinker/current drinker/former drinker), passive smoking (yes/no), family income (<100,000 yuan/100,000–200,000 yuan/≥200,000 yuan), and abstinence time (continuous). Confounding variable identification was based on prior knowledge or biological plausibility. In addition, we performed analyses separately for age (<35 and ≥35 years), BMI (<24 and ≥24), family income (<100,000 yuan and ≥100,000 yuan), passive smoking (no and yes), and alcohol drinking (never and ever), considering the possibility that smoking may impact semen quality differentially by these variates.

All statistical analyses were performed using R software version 3.4.1 (R Core Team R, 2016). All statistical tests were two-tailed and *P* value less than 0.05 was considered statistically significant. To address multiple testing, we also calculated the false discovery rate (FDR) using the Benjamini–Hochberg (BH) procedure.

## Results

Participant characteristics by smoking status are presented in Table 1. Among the 1631 participants studied, 1011 (62.0%) were never smokers and 620 (38%) were ever smokers including 534 (32.7%) current smokers and 86 (5.3%) former smokers. Compared with never smokers, ever smokers had higher BMI, were more likely to be drinkers, and had lower education attainment and lower family income (Table 1). Among the 620 ever smokers, the intensity of smoking in 285 (46%) men were <10 cigarette/day, 263 (42.4%) were 10–20, and 72 (11.6%) were ≥ 20. Most of men (388, 62.6%) smoked <5 pack-years (eTable 1; http://links.lww.com/EE/A48). The mean number of pack-years was 4.76 and the majority of men were 20–25 years old at initiation of smoking (58.2%; eTable 1; http://links.lww.com/EE/A48). The average values of the 14 semen quality parameters among the study participant are shown in eTable 2 (http://links.lww.com/EE/A48).

**TABLE 1.**
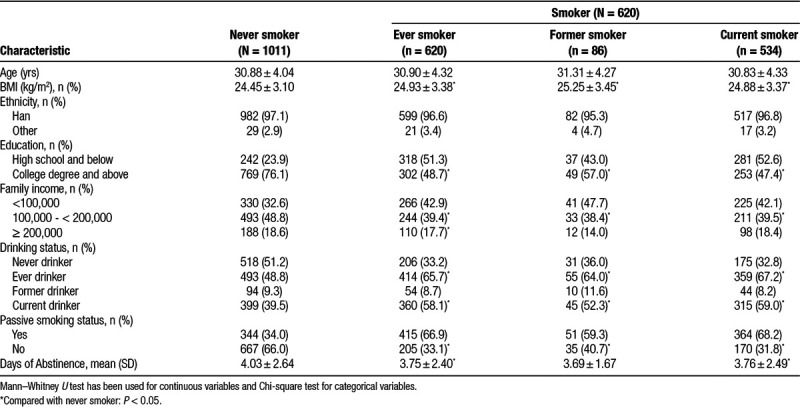
Baseline characteristics by smoking status.

## Smoking status in relation to semen parameters

In unadjusted models, statistically significant associations were observed between ever smoking and semen volume, sperm concentration, total sperm count, VCL, and VAP (for semen volume: *β* = −0.05, *P* < 0.001; for sperm concentration: *β* = −4.15, *P* = 0.046; for total sperm count: *β* = −0.28, *P* < 0.001; for VCL: *β* = 1.13, *P* = 0.046; for VSL: *β* = 0.76, *P* = 0.046; eTable 3; http://links.lww.com/EE/A48). However, when all the potential confounders were included in the multivariate models, there were no significant differences. These results were similar when former smokers and current smokers were each compared with never smokers (eTable 3; http://links.lww.com/EE/A48).

## Semen quality in men with different intensity of smoking and smoking habit duration

Compared with never smokers, higher smoking intensity (≥20 cigarettes smoked daily) was related to lower semen volume and total sperm count (for semen volume: multivariable-adjusted *β* = −0.08, *P* = 0.037; for total sperm count: multivariable-adjusted *β* = −0.45, *P* = 0.043; Table 2). No significant associations were detected with other semen quality parameters (Table 2). No significant associations were found with any subgroup of smoking duration and semen quality in multivariable models compared with never smokers (eTable 4; http://links.lww.com/EE/A48).

**TABLE 2.**
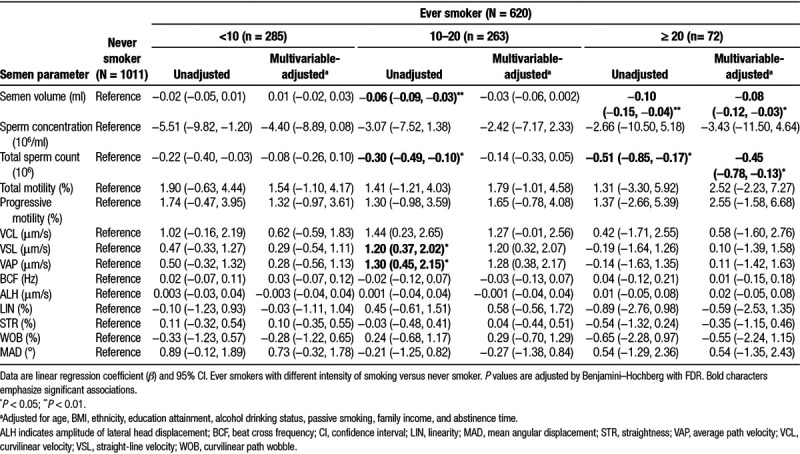
Comparison of semen parameters in never smokers and ever smokers with different intensity of smoking (number of cigarettes smoked daily).

## Semen quality in men with different cumulative dose of smoking

Results of linear regression models of cumulative dose of smoking (pack-years) in association with semen quality are shown in Table 3. In multivariable-adjusted analysis, only the highest category of cumulative doses of smoking (≥10 pack-years) was significantly negatively associated with semen volume (multivariable-adjusted *β* = −0.10, *P* = 0.001) and total sperm count (multivariable-adjusted *β* = −0.42, *P* = 0.037). Positive associations were observed only in multivariable-adjusted analyses for total motility (*β* = 6.02, *P* = 0.037) and progressive motility (*β* = 5.52, *P* = 0.037; Table 3). When ever smokers were divided back into former smokers and current smokers, the significant associations between cumulative smoking and semen quality were only found in the much larger group of current smokers (for semen volume: *β* = −0.08, *P* = 0.011; for total motility: *β* = 6.77, *P* = 0.027; for progressive motility: *β* = 6.11, *P* = 0.027; eTable 5; http://links.lww.com/EE/A48) and were not seen in the 86 former smokers (eTable 6; http://links.lww.com/EE/A48).

**TABLE 3.**
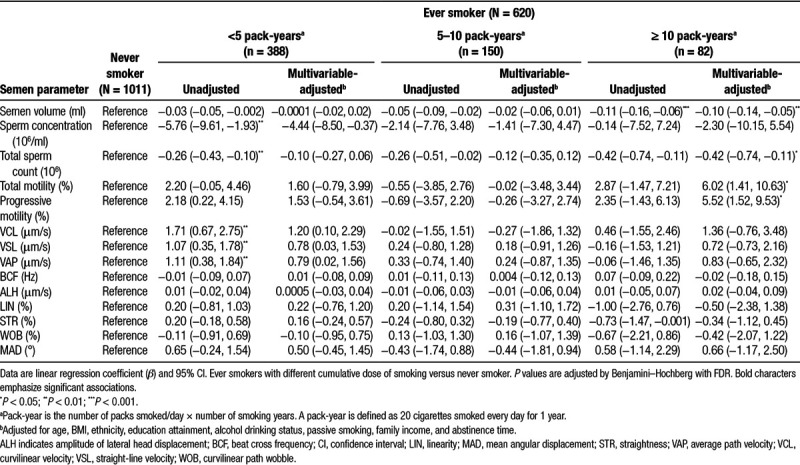
Comparison of semen parameters in never smokers and smokers with different cumulative dose of smoking.

## Semen quality in men with different age at smoking initiation and different years of quit smoking

Because the duration of smoking was related to semen parameters, we investigated the role of the age of smoking initiation. Younger ages of initiation were associated with semen volume only in unadjusted models (Table 4); no significant associations were found between any semen quality parameters and age at smoking initiation in the multivariable models (Table 4). Additionally, investigating former smokers with different durations of cessation and semen quality, we did not find any significant associations in the multivariable models (eTable 7; http://links.lww.com/EE/A48).

**TABLE 4.**
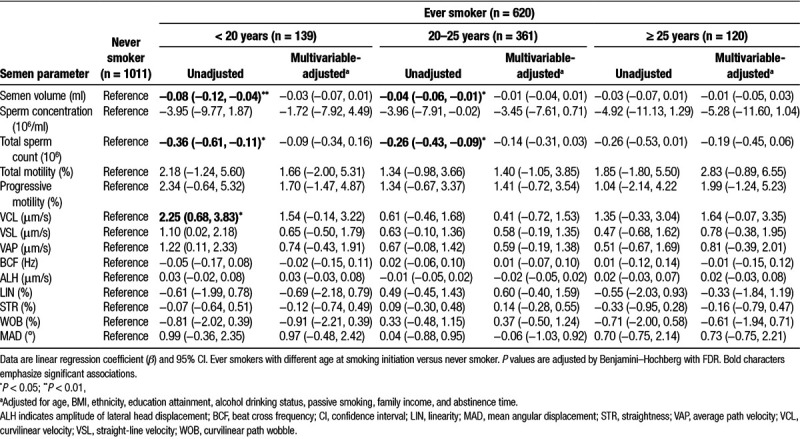
Comparison of semen parameters in never smokers and smokers with different age at smoking initiation.

## Dose–response relation between smoking and semen quality

We observed a negative dose-dependent association between cumulative doses of cigarette smoking (pack-years) and semen volume (Table 5). The coefficient increased gradually across the categories and was −0.10 for semen volume (*P*, trend <0.001) and −0.42 for total sperm count (*P*, trend = 0.010; Table 5). Additionally, positive dose-dependent associations were also found between pack-years and both total motility (*P*, trend < 0.032) and progressive motility (*P*, trend = 0.028; eTable 8; http://links.lww.com/EE/A48).

**TABLE 5.**
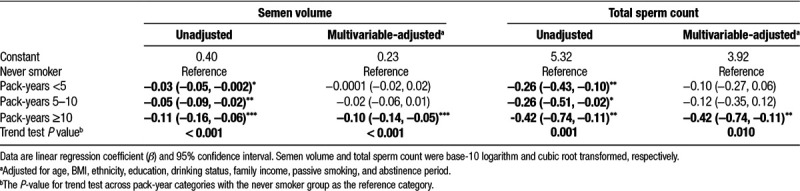
Association between smoking and semen quality shows a dose–response pattern with increased pack-years.

## Stratification analysis of association between smoking and semen quality

The results of the analyses of smoking in relation to semen volume and total sperm motility stratified by age, BMI, family income, passive smoking, and alcohol drinking are summarized in eTables 9 and 10 (http://links.lww.com/EE/A48). We found no indication that the associations varied by age (*P* for interaction = 0.117), BMI (*P* for interaction = 0.433), and passive smoking (*P* for interaction = 0.557). There was a suggestion of a stronger inverse association in lower family income (*P* for interaction = 0.011) and never drinkers (*P* for interaction = 0.022; eTable 9; http://links.lww.com/EE/A48). There was no evidence that associations between higher cumulative dose of smoking and total sperm count differed by age, BMI, family income, passive smoking, or alcohol drinking (eTable 10; http://links.lww.com/EE/A48).

## Discussion

In the present study, that in a large sample size of males with known fertility, heavy cigarette smoking was associated with decreased semen volume and total sperm count and increased total motility and progressive motility after adjusting for potential confounding variables. Semen quality was not significantly different between former smokers and never smokers. No significant association was found between smoking and sperm motion parameters. It is known that sperm development takes approximately 3 months. This result implied that smoking may not affect the development of sperm, but mainly affect the early stage of spermatogenesis.

Complete assessment of the smoking impact on semen quality requires a separate and combined analysis of cumulative effects of exposure duration and dose. Smoking intensity and duration mainly reflect exposure dose and exposure lasting time, respectively. Therefore, in our present study, we classified smoking exposure according to smoking duration, smoking intensity, and cumulative exposure (pack-years). Cigarette contains many compounds that are known as chemical carcinogens and mutagens in humans. While acute exposures of highly toxic substances of smoking could cause dramatic short- and long-term changes in semen parameters, these exposures are relatively rare in the population. Chronic, low-dose exposures may not have as profound effects as acute exposures. The sustained nature of smoking exposures, however, could contribute to clinically significant impairments of spermatogenesis, reflected in alternations in semen parameters.

In our analyses of cumulative dose of smoking (pack-years), we found a significant decrease in semen volume and total sperm count among ever smokers with higher cumulative doses of smoking. A previous meta-analysis by Li et al^15^ reported that smokers had lower semen volume and total sperm count when compared with never smokers. However, a recent meta-analysis by Sharma et al^16^ reported decreased sperm count in smokers compared with nonsmokers but semen volume was not affected. Another study found that current smokers showed significantly lower ultrasound-derived seminal vesicle volumes, either before or after ejaculation.^19^ A study conducted among 243 fertile males found that cigarette smoking was associated with significantly lower semen volumes.^20^

Our findings of positive associations between smoking and motility are in accordance with animal studies. Dai et al^21^ found that nicotine-treated mice have elevated sperm motility parameters, consistent with our finding that heavy smoking (≥10 pack-years) was associated with higher total motility and progressive motility. The effect of age at smoking initiation on semen quality has not previously been investigated. We did not find age to be significantly associated with semen quality after adjusted for confounding variables. However, in this population, the range of ages of initiation was narrow: we had very few individuals who began smoking before puberty, an important period for maturation of the testis that could influence later sperm quality.

In the current study, no significant difference in semen quality was found in any category of former smokers compared with never smokers. This result suggests that smoking cessation might have a restorative effect on semen quality. Two previous studies that followed men up to 12 months after smoking cessation found that semen quality was markedly improved.^22,23^ Sperm development takes approximately 70–90 days in humans and all of our former smokers had quit more than 6 months before semen collection. We speculate that the lack of significant associations between former smoking and semen quality in our study may be attributed to the lack of smoking exposure during sperm development. It is possible that men with below-average semen quality, who wish to have children, might especially benefit from quitting smoking.

Semen quality is strongly correlated with fecundity.^24^ Although all the participants in our study were fertile males, their partners may vary in time-to-pregnancy (TTP). It can be speculated that lower semen quality may lead to longer TTP, and previous studies identified longer TTP in partners of male smokers.^25,26^ Additionally, it has been demonstrated that tobacco smoking is associated with increases in DNA damage, mutations, and epigenetic abnormalities in sperm which could in turn impact sperm parameters.^27–29^ Paternal smoking can lead to increased susceptibility to several diseases in offspring.^19,29^ Therefore, poor sperm quality and quantity might be an important mediator in the association between paternal smoking and offspring health.

Previous studies reported that cigarette smoking can cause chromosomal aberration, DNA damage, and alter the DNA methylation pattern in sperms.^30,31^ Several mechanisms have been hypothesized. These mechanisms were concerning the deterioration of spermatogenesis, induction of ultrastructural abnormalities, apoptosis, and abnormal epigenetic modification. Though some males exhibit normal semen quality, the sperm may harbor some abnormalities, such as aberrant DNA methylation and abnormal expression of noncoding RNA. Therefore, it is possible that smoking may influence fecundability by affecting sperm epigenetic modifications, sperm chromatin damage, and without volume, count, and motility as mediators. Future studies are needed to illustrate the underlying molecular mechanism.

This study has limitations that deserve mention. Smoking information was self-reported. However, previous studies have shown that the accuracy between self-reported and biochemical elements in the blood is high,^32^ and self-reporting tend to underestimate the amount of smoking.^33^ Another important limitation was that only one semen sample was analyzed for semen quality for each participant. However, a previous study reported that a single semen sample may suffice for identifying differences in semen quality between groups of men.^34^ As in any epidemiologic study, although we adjusted for many covariates, unmeasured confounding is possible.

A major strength of this study is the large well-characterized cohort of fertile men. The number of participants in the earlier studies investigating the association between smoking and semen quality of fertile men is 889 participants^35^ and most have been much smaller. Secondly, we assessed a wide range of 14 semen parameters to investigate the associations between smoking and semen quality in a more comprehensive manner than in most studies. Thirdly, we conducted smoking status subanalyses, such as evaluating current versus former smoking. We also examined smoking history in greater detail than in many previous studies considering the amount smoked, duration, pack-years, and age at initiation. Fourthly, we took great effort to obtain information on lifestyle factors and environmental exposures for all participants and adjusted for several possible confounders, such as age, BMI, ethnicity, education, drinking status, family income, and abstinence period. We also analyzed the association between smoking and semen quality stratified by age, BMI, passive smoking, etc. The larger size, consideration of potential confounders and more comprehensive evaluation of both smoking variables and sperm parameters based on WHO guidelines enabled statistically robust analyses of the impact of this common lifestyle exposure and sperm quality.

## Conclusions

Cigarette smoking was associated with lower semen volume and total sperm count and higher sperm motility in fertile men, especially among those who are heavy smokers. The detrimental effects of smoking on semen quality were not seen in men who had stopped smoking. This work has implications for public health research to help shape opinions related to smoking and male fertility and may shed light on how environmental exposures can impact the development of abnormal semen quality.

## Supplementary Material
