# Supplementary material for: Semen quality and cigarette smoking in a cohort of healthy fertile men
Source: Environ Epidemiol. 2019 Jun 25;3(4):e055. doi: 10.1097/EE9.0000000000000055 (PMC6693933; doi:10.1097/EE9.0000000000000055)
Supplement: Supplementary file 1 [file ee9-3-e055-s001.docx]

**Supplementary Materials**

**Table S1.** Smoking habit in ever smokers.

| **Characteristic** | **Ever smoker (n = 620)** | **Former smoker (n = 86)** | **Current smoker (n =534)** |
| --- | --- | --- | --- |
| **Intensity of smoking** |  |  |  |
| < 10 | 285 (46.0) | 46 (53.5) | 239 (44.8) |
| 10-20 | 263 (42.4) | 26 (30.2) | 237 (44.4) |
| ≥ 20 | 72 (11.6) | 14 (16.3) | 58 (10.9) |
| Mean (SD) | 9.24 ± 5.87 | 9.69 ± 6.25 | 9.17 ± 5.81 |
| **Smoking habit duration** |  |  |  |
| < 5 | 91 (14.7) | 25 (29.1) | 66 (12.4) |
| 5-10 | 269 (43.4) | 34 (39.5) | 235 (44.0) |
| ≥ 10 | 260 (41.9) | 27 (31.4) | 233 (43.6) |
| Mean (SD) | 9.20 ± 5.01 | 7.66 ± 4.82 | 9.45 ± 5.00 |
| **Cumulative dose of smoking** |  |  |  |
| < 5 pack-years | 388 (62.6) | 58 (67.4) | 330 (61.8) |
| 5-10 pack-years | 150 (24.2) | 20 (23.3) | 130 (24.3) |
| ≥ 10 pack-years | 82 (13.2) | 8 (9.3) | 74 (13.9) |
| Mean (SD) | 4.76 ± 4.76 | 3.92 ± 3.81 | 4.90 ± 4.89 |
| **Age at smoking initiation** |  |  |  |
| < 20 | 139 (22.4) | 23 (26.7) | 116 (21.7) |
| 20-25 | 361 (58.2) | 44 (51.2) | 317 (59.4) |
| ≥ 25 | 120 (19.4) | 19 (22.1) | 101 (18.9) |
| Mean (SD) | 21.45 ± 3.35 | 21.23 ± 3.90 | 21.49 ± 3.26 |

SD: standard deviation.

**Table S2.** The average values of the 14 semen quality parameters among the study participants.

| **Semen parameter^a^** | **Mean ± SD** |
| --- | --- |
| **Semen volume (ml)** | 2.72 ± 1.32 |
| **Sperm concentration (10^6^/ml)** | 62.25 ± 32.82 |
| **Total sperm count (10^6^)** | 173.58 ± 134.48 |
| **Total motility (%)** | 55.20 ± 19.27 |
| **Progressive motility (%)** | 43.87 ± 16.81 |
| **VCL (μm/s)** | 47.92 ± 8.91 |
| **VSL (μm/s)** | 29.96 ± 6.09 |
| **VAP (μm/s)** | 33.67 ± 6.25 |
| **BCF (Hz)** | 5.10 ± 0.69 |
| **ALH (μm/s)** | 3.64 ± 1.06 |
| **LIN (%)** | 62.58 ± 7.81 |
| **STR (%)** | 88.66 ± 3.26 |
| **WOB (%)** | 70.45 ± 6.81 |
| **MAD (°)** | 56.71 ± 7.62 |

^a^ALH, amplitude of lateral head displacement; BCF, beat cross frequency; LIN, linearity; MAD, mean angular displacement; STR, straightness; VAP, average path velocity; VCL, curvilinear velocity; VSL, straight line velocity; WOB, curvilinear path wobble.

**Table S3.** Coefficients from linear regression for smoking status in relation to semen parameters.

| **Semen parameter^a^** | **Never smoker** **(n =** **1011)** | **Smoker (n = 620)** | | | | | |
| --- | --- | --- | --- | --- | --- | --- | --- |
|  |  | **Ever smoker (n = 620)** | | **Former smoker (n = 86)** | | **Current smoker (n = 534)** | |
|  |  | **Unadjusted** | **Multivariable-adjusted^b^** | **Unadjusted** | **Multivariable-adjusted^b^** | **Unadjusted** | **Multivariable-adjusted^b^** |
| **Semen volume (ml)** | Reference | **-0.05 (-0.07, -0.02)^***^** | -0.02 (-0.04, 0.01) | -0.04 (-0.09, 0.004) | -0.01 (-0.05, 0.47) | **-0.05 (-0.07, -0.02)^**^** | -0.02 (-0.04, 0.01) |
| **Sperm concentration (10^6^/ml)** | Reference | **-4.15 (-7.42, -0.87)^*^** | -3.50 (-7.09, 0.10) | -7.94 (-15.35, -0.53) | -6.62 (-14.15, 0.91) | -3.53 (-6.97, 0.10) | -2.76 (-6.55, 1.02) |
| **Total sperm count (10^6^)** | Reference | **-0.28 (-0.43, -0.14)^***^** | -0.14 (-0.29, 0.002) | -0.41 (-0.72, -0.10) | -0.23 (-0.53, 0.07) | **-0.26 (-0.41, -0.12)^**^** | -0.12 (-0.27, 0.03) |
| **Total motility (%)** | Reference | 1.63 (-0.30, 3.55) | 1.74 (-0.37, 3.86) | -0.75 (-5.03, 3.54) | -0.52 (-4.84, 3.80) | 2.01 (-0.01, 4.02) | 2.17 (-0.06, 4.39) |
| **Progressive motility (%)** | Reference | 1.51 (-0.17, 3.19) | 1.59 (-0.25, 3.42) | -0.46 (-4.16, 3.24) | -0.29 (-4.01, 3.43) | 1.83 (0.07, 3.58) | 1.94 (0.01, 3.87) |
| **VCL (μm/s)** | Reference | **1.13 (0.23, 2.02)^*^** | 0.88 (-0.09, 1.85) | 0.64 (-1.31, 2.58) | 0.29 (-1.67, 2.25) | **1.20 (0.27, 2.14)^*^** | 0.97 (-0.05, 1.99) |
| **VSL (μm/s)** | Reference | 0.70 (-0.10, 1.31) | 0.63 (-0.03, 1.30) | -0.03 (-1.35, 1.29) | -0.20 (-1.53, 1.13) | **0.82 (0.19, 1.46)^*^** | 0.77 (0.08, 1.47) |
| **VAP (μm/s)** | Reference | **0.76 (-0.14, 1.39)^*^** | 0.67 (-0.01, 1.35) | 0.07 (-1.27, 1.41) | -0.14 (-1.49, 1.20) | **0.88 (0.22, 1.53)^*^** | 0.80 (0.08, 1.51) |
| **BCF (Hz)** | Reference | -0.004 (-0.06, 0.07) | 0.002 (-0.07, 0.08) | 0.04 (-0.11, 0.19) | 0.05 (-0.10, 0.19) | -0.002 (-0.07, 0.07) | -0.01 (-0.09, 0.07) |
| **ALH (μm/s)** | Reference | 0.003 (-0.02, 0.03) | -0.004 (-0.03, 0.03) | 0.01 (-0.05, 0.07) | 0.01 (-0.05, 0.07) | 0.002 (-0.03, 0.03) | -0.001 (-0.03, 0.03) |
| **LIN (%)** | Reference | 0.04 (-0.74, 0.82) | 0.16 (-0.71, 1.02) | -0.86 (-2.60, 0.89) | -0.85 (-2.63, 0.93) | 0.19 (-0.63, 1.01) | 0.34 (-0.57, 1.25) |
| **STR (%)** | Reference | -0.03 (-0.35, 0.30) | 0.03 (-0.33, 0.38) | -0.37 (-1.04, 0.30) | -0.04 (-1.06, 0.30) | 0.03 (-0.31, 0.37) | 0.10 (-0.28, 0.47) |
| **WOB (%)** | Reference | -0.13 (-0.81, 0.56) | -0.08 (-0.83, 0.68) | -0.79 (-2.27, 0.70) | -0.85 (-2.37, 0.67) | -0.02 (-0.73, 0.70) | 0.05 (-0.75, 0.84) |
| **MAD (°)** | Reference | 0.38 (-0.38, 1.14) | 0.30 (-0.54, 1.14) | 1.07 (-0.63, 2.78) | 1.05 (-0.69, 2.79) | 0.27 (-0.54, 1.07) | 0.21 (-0.68, 1.10) |

Data are linear regression coefficient (β) and 95%CI. Ever smoker, former smoker or current smoker versus never smoker: ^*^*P* < 0.05; ^**^*P* < 0.01; ^***^*P* < 0.001. *P*-values are adjusted by Benjamini-Hochberg with FDR. Bold characters emphasize significant associations.

^a^ALH, amplitude of lateral head displacement; BCF, beat cross frequency; LIN, linearity; MAD, mean angular displacement; STR, straightness; VAP, average path velocity; VCL, curvilinear velocity; VSL, straight line velocity; WOB, curvilinear path wobble.

^b^Adjusted for age, BMI, ethnicity, education attainment, alcohol drinking status, passive smoking, family income, and abstinence time.

**Table S4.** Comparison of semen parameters in never smokers and ever smokers with different smoking habit duration (years).

| **Semen parameter^a^** | **Never smoker (n = 1011)** | **Ever smoker (n = 620)** | | | | | |
| --- | --- | --- | --- | --- | --- | --- | --- |
|  |  | **< 5 (n = 91)** | | **5-10 (n = 269)** | | **≥ 10 (n = 260)** | |
|  |  | **Unadjusted** | **Multivariable-adjusted^b^** | **Unadjusted** | **Multivariable-adjusted^b^** | **Unadjusted** | **Multivariable-adjusted^b^** |
| **Semen volume (ml)** | Reference | -0.01 (-0.06, 0.04) | 0.002 (-0.04, 0.05) | **-0.05 (-0.08, -0.02)****^**^** | -0.02 (-0.05, 0.01) | **-0.05 (-0.08, -0.02)****^*^** | -0.02 (-0.05, 0.01) |
| **Sperm concentration (10^6^/ml)** | Reference | -7.53 (-14.56, -0.50) | -6.20 (-13.36, 0.96) | **-6.07 (-10.47, -1.66)^*^** | -4.39 (-9.04, 0.26) | -0.97 (-5.43, 3.49) | -1.36 (-6.29, 3.56) |
| **Total sperm count (10^6^)** | Reference | -0.25 (-0.55, 0.05) | -0.16 (-0.45, 0.13) | **-0.36 (-0.55, -0.17)^**^** | -0.18 (-0.37, 0.005) | -0.22 (-0.41, -0.02) | -0.09 (-0.29, 0.11) |
| **Total motility (%)** | Reference | 2.19 (-1.95, 6.32) | 0.62 (-3.59, 4.83) | 1.53 (-1.06, 4.13) | 0.71 (-2.03, 3.44) | 1.53 (-1.10, 4.16) | 3.38 (0.48, 6.28) |
| **Progressive motility (%)** | Reference | 2.65 (-0.96, 6.26) | 1.05 (-2.61, 4.71) | 1.48 (-0.78, 3.74) | 0.58 (-1.80, 2.95) | 1.14 (-1.15, 3.43) | 2.94 (0.43, 5.46) |
| **VCL (μm/s)** | Reference | 1.63 (-0.28, 3.54) | 0.79 (-1.15, 2.73) | 1.04 (-0.16, 2.24) | 0.40 (-0.86, 1.65) | 1.03 (-0.18, 2.25) | 1.46 (0.13, 2.79) |
| **VSL (μm/s)** | Reference | 0.90 (-0.41, 2.21) | 0.35 (-0.97, 1.67) | 0.83 (0.02, 1.65) | 0.43 (-0.42, 1.29) | 0.50 (-0.33, 1.33) | 0.98 (0.07, 1.89) |
| **VAP (μm/s)** | Reference | 0.91 (-0.43, 2.25) | 0.30 (-1.06, 1.65) | 0.96 (0.13, 1.80) | 0.53 (-0.34, 1.41) | 0.51 (-0.35, 1.36) | 0.97 (0.04, 1.90) |
| **BCF (Hz)** | Reference | -0.04 (-0.18, 0.11) | 0.002 (-0.15, 0.15) | 0.004 (-0.09, 0.10) | 0.03 (-0.07, 0.13) | 0.02 (-0.08, 0.11) | -0.03 (-0.13, 0.08) |
| **ALH (μm/s)** | Reference | 0.02 (-0.04, 0.08) | 0.004 (-0.06, 0.06) | -0.01 (-0.05, 0.03) | -0.02 (-0.06, 0.02) | 0.01 (-0.03, 0.05) | 0.02 (-0.02, 0.06) |
| **LIN (%)** | Reference | 0.07 (-1.61, 1.74) | -0.002 (-1.72, 1.72) | 0.36 (-0.70, 1.41) | 0.31 (-0.81, 1.43) | -0.29 (-1.36, 0.78) | 0.05 (-1.13, 1.23) |
| **STR (%)** | Reference | 0.38 (-0.32, 1.08) | 0.23 (-0.49, 0.94) | 0.05 (-0.39, 0.48) | -0.04 (-0.50, 0.43) | -0.25 (-0.69, 0.20) | 0.01 (-0.48, 0.51) |
| **WOB (%)** | Reference | -0.29 (-1.75, 1.17) | -0.33 (-1.83, 1.17) | 0.27 (-0.65, 1.18) | 0.25 (-0.73, 1.22) | -0.47 (-1.40, 0.45) | -0.34 (-1.37, 0.70) |
| **MAD (°)** | Reference | 0.74 (-0.90, 2.38) | 0.53 (-1.15, 2.21) | 0.09 (-0.94, 1.11) | -0.06 (-1.15, 1.03) | 0.55 (-0.49, 1.59) | 0.62 (-0.54, 1.77) |

Data are linear regression coefficient (β) and 95%CI. Ever smokers with different smoking habit duration versus never smoker: ^*^*P* < 0.05; ^**^*P* < 0.01. *P*-values are adjusted by Benjamini-Hochberg with FDR. Bold characters emphasize significant associations.

^a^ALH, amplitude of lateral head displacement; BCF, beat cross frequency; LIN, linearity; MAD, mean angular displacement; STR, straightness; VAP, average path velocity; VCL, curvilinear velocity; VSL, straight line velocity; WOB, curvilinear path wobble.

^b^Adjusted for age, BMI, ethnicity, education attainment, alcohol drinking status, passive smoking, family income, and abstinence time.

**Table S5.** Comparison of semen parameters in never smokers and current smokers with different cumulative dose of smoking.

| **Semen parameter^a^** | **Never smoker (n = 1011)** | **Current smoker (n = 534)** | | | | | |
| --- | --- | --- | --- | --- | --- | --- | --- |
|  |  | **< 5 pack-years^b^ (n = 330)** | | **5-10 pack-years^b^ (n = 130)** | | **≥ 10 pack-years^b^ (n = 74)** | |
|  |  | **Unadjusted** | **Multivariable-adjusted^c^** | **Unadjusted** | **Multivariable-adjusted^c^** | **Unadjusted** | **Multivariable-adjusted^c^** |
| **Semen volume (ml)** | Reference | **-0.03 (-0.06, -0.01)^*^** | -0.004 (-0.03, 0.02) | -0.04 (-0.08, -0.004) | -0.02 (-0.05, 0.02) | **-0.10 (-0.15, -0.05)****^**^** | **-0.08 (-0.13, -0.04)^*^** |
| **Sperm concentration (10^6^/ml)** | Reference | **-5.23 (-9.30, -1.16)^*^** | -3.59 (-7.93, 0.76) | -1.63 (-7.61, 4.35) | -1.10 (-7.35, 5.15) | 0.67 (-7.06, 8.40) | -1.66 (-9.85, 6.53) |
| **Total sperm count (10^6^)** | Reference | **-0.25 (-0.43, -0.08)^*^** | -0.07 (-0.25, 0.10) | -0.23 (-0.48, 0.03) | -0.10 (-0.35, 0.15) | -0.38 (-0.72, -0.05) | -0.39 (-0.72, -0.07) |
| **Total motility (%)** | Reference | 2.41 (0.03, 4.80) | 1.75 (-0.80, 4.29) | 0.16 (-3.35, 3.67) | 0.84 (-2.82, 4.50) | 3.44 (-1.10, 7.97) | **6.77 (1.97, 11.57)^*^** |
| **Progressive motility (%)** | Reference | 2.30 (0.21, 4.38) | 1.58 (-0.63, 3.79) | 0.10 (-2.95, 3.16) | 0.68 (-2.50, 3.86) | 2.77 (-1.18, 6.72) | **6.11 (1.95, 10.28)^*^** |
| **VCL (μm/s)** | Reference | **1.87 (0.77, 2.97)^**^** | 1.30 (0.13, 2.47) | 0.06 (-1.56, 1.68) | -0.08 (-1.76, 1.61) | 0.24 (-1.85, 2.34) | 1.23 (-0.98, 3.44) |
| **VSL (μm/s)** | Reference | **1.23 (0.48, 1.98)^**^** | 0.92 (0.12, 1.72) | 0.47 (-0.63, 1.58) | 0.49 (-0.66, 1.63) | -0.38 (-1.81, 1.05) | 0.58 (-0.93, 2.08) |
| **VAP (μm/s)** | Reference | **1.27 (0.49, 2.03)^**^** | 0.92 (0.10, 1.73) | 0.54 (-0.60, 1.67) | 0.52 (-0.66, 1.69) | -0.26 (-1.73, 1.20) | 0.71 (-0.84, 2.25) |
| **BCF (Hz)** | Reference | -0.01 (-0.10, 0.07) | 0.002 (-0.09, 0.09) | -0.01 (-0.14, 0.11) | -0.02 (-0.15, 0.11) | 0.07 (-0.10, 0.23) | -0.03 (-0.20, 0.14) |
| **ALH (μm/s)** | Reference | 0.01 (-0.03, 0.04) | -0.002 (-0.04, 0.04) | -0.01 (-0.07, 0.04) | -0.01 (-0.07, 0.04) | 0.01 (-0.06, 0.08) | 0.02 (-0.05, 0.09) |
| **LIN (%)** | Reference | 0.40 (-0.57, 1.37) | 0.45 (-0.60, 1.49) | 0.44 (-0.99, 1.87) | 0.58 (-0.93, 2.08) | -1.20 (-3.05, 0.65) | -0.65 (-2.62, 1.32) |
| **STR (%)** | Reference | 0.29 (-0.11, 0.69) | 0.26 (-0.17, 0.69) | -0.10 (-0.69, 0.49) | -0.02 (-0.64, 0.60) | -0.91 (-1.68, -0.15) | -0.50 (-1.31, 0.31) |
| **WOB (%)** | Reference | 0.04 (-0.81, 0.88) | 0.07 (-0.84, 0.98) | 0.28 (-0.97, 1.52) | 0.29 (-1.02, 1.61) | -0.78 (-2.39, 0.84) | -0.52 (-2.24, 1.21) |
| **MAD (°)** | Reference | 0.54 (-0.41, 1.49) | 0.38 (-0.65, 1.40) | -0.60 (-2.00, 0.80) | -0.51 (-1.98, 0.96) | 0.55 (-1.26, 2.36) | 0.70 (-1.22, 2.63) |

Data are linear regression coefficient (β) and 95%CI. Current smokers with different cumulative dose of smoking versus never smoker: ^*^*P* < 0.05; ^**^*P* < 0.01. *P*-values are adjusted by Benjamini-Hochberg with FDR. Bold characters emphasize significant associations.

^a^ALH, amplitude of lateral head displacement; BCF, beat cross frequency; LIN, linearity; MAD, mean angular displacement; STR, straightness; VAP, average path velocity; VCL, curvilinear velocity; VSL, straight line velocity; WOB, curvilinear path wobble.

^b^Pack-year is the number of packs smoked/day × number of smoking years. A pack-year is defined as twenty cigarettes smoked every day for one year.

^c^Adjusted for age, BMI, ethnicity, education attainment, alcohol drinking status, passive smoking, family income, and abstinence time.

**Table S6.** Comparison of semen parameters in never smokers and former smokers with different cumulative dose of smoking.

| **Semen parameter^a^** | **Never smoker (n = 1011)** | **Former smoker (n = 86)** | | | | | |
| --- | --- | --- | --- | --- | --- | --- | --- |
|  |  | **< 5 pack-years^b^ (n = 58)** | | **5-10 pack-years^b^ (n = 20)** | | **≥ 10 pack-years^b^ (n = 8)** | |
|  |  | **Unadjusted** | **Multivariable-adjusted^c^** | **Unadjusted** | **Multivariable-adjusted^c^** | **Unadjusted** | **Multivariable-adjusted^c^** |
| **Semen volume (ml)** | Reference | 0.003 (-0.05, 0.06) | 0.02 (-0.03, 0.07) | -0.11 (-0.21, -0.01) | -0.04 (-0.13, 0.04) | **-0.23 (-0.38, -0.08)^*^** | -0.17 (-0.31, -0.04) |
| **Sperm concentration (10^6^/ml)** | Reference | -8.84 (-17.75, 0.07) | -8.20 (-0.17, 0.72) | -5.45 (-20.36, 9.45) | -1.95 (-0.17, 13.14) | -7.60 (-31.02, 15.83) | -6.27 (-0.30, 17.58) |
| **Total sperm count (10^6^)** | Reference | -0.32 (-0.70, 0.05) | -0.24 (-0.59, 0.11) | -0.51 (-1.14, 0.12) | -0.09 (-0.69, 0.50) | -0.78 (-1.77, 0.21) | -0.55 (-1.49, 0.39) |
| **Total motility (%)** | Reference | 1.00 (-4.15, 6.15) | 0.80 (-4.31, 5.91) | -5.15 (-13.76, 3.46) | -5.43 (-14.08, 3.22) | -2.39 (-15.93, 11.15) | 1.81 (-11.86, 15.49) |
| **Progressive motility (%)** | Reference | 1.54 (-2.91, 5.98) | 1.37 (-3.03, 5.76) | -5.82 (-13.26, 1.62) | -6.28 (-13.72, 1.16) | -1.56 (-13.25, 10.13) | 2.20 (-9.55, 13.96) |
| **VCL (μm/s)** | Reference | 0.79 (-1.55, 3.13) | 0.52 (-1.80, 2.84) | -0.52 (-4.44, 3.39) | -1.36 (-5.29, 2.57) | 2.44 (-3.71, 8.59) | 2.80 (-3.41, 9.01) |
| **VSL (μm/s)** | Reference | 0.14 (-1.45, 1.73) | 0.03 (-1.54, 1.60) | -1.29 (-3.95, 1.37) | -1.86 (-4.51, 0.80) | 1.89 (-2.29, 6.07) | 2.33 (-1.87, 6.53) |
| **VAP (μm/s)** | Reference | 0.20 (-1.41, 1.82) | 0.05 (-1.54, 1.64) | -1.02 (-3.72, 1.67) | -1.62 (-4.32, 1.07) | 1.85 (-2.39, 6.09) | 2.17 (-2.09, 6.43) |
| **BCF (Hz)** | Reference | 0.01 (-0.17, 0.18) | 0.01 (-0.17, 0.19) | 0.13 (-0.16, 0.43) | 0.16 (-0.14, 0.46) | 0.06 (-0.41, 0.52) | 0.04 (-0.43, 0.51) |
| **ALH (μm/s)** | Reference | 0.01 (-0.06, 0.09) | 0.01 (-0.07, 0.08) | -0.003 (-0.13, 0.12) | -0.002 (-0.13, 0.12) | 0.03 (-0.17, 0.22) | 0.03 (-0.16, 0.23) |
| **LIN (%)** | Reference | -0.93 (-3.02, 1.17) | -0.85 (-2.96, 1.26) | -1.35 (-4.85, 2.16) | -1.58 (-5.14, 1.99) | 0.88 (-4.63, 6.39) | 1.06 (-4.58, 6.69) |
| **STR (%)** | Reference | -0.29 (-1.09, 0.52) | -0.28 (-1.09, 0.53) | -1.14 (-2.49, 0.20) | -1.28 (-2.64, 0.09) | 0.93 (-1.19, 3.04) | 1.14 (-1.01, 3.30) |
| **WOB (%)** | Reference | -0.93 (-2.72, 0.85) | -0.91 (-2.71, 0.88) | -0.80 (-3.78, 2.19) | -1.00 (-4.05, 2.04) | 0.27 (-4.42, 4.97) | 0.10 (-4.71, 4.91) |
| **MAD (°)** | Reference | 1.25 (-0.80, 3.30) | 1.17 (-0.89, 3.23) | 0.67 (-2.75, 4.09) | 0.70 (-2.79, 4.19) | 0.80 (-4.59, 6.18) | 1.00 (-4.51, 6.52) |

Data are linear regression coefficient (β) and 95%CI. Former smokers with different cumulative dose of smoking versus never smoker: ^*^*P* < 0.05. *P*-values are adjusted by Benjamini-Hochberg with FDR. Bold characters emphasize significant associations.

^a^ALH, amplitude of lateral head displacement; BCF, beat cross frequency; LIN, linearity; MAD, mean angular displacement; STR, straightness; VAP, average path velocity; VCL, curvilinear velocity; VSL, straight line velocity; WOB, curvilinear path wobble.

^b^Pack-year is the number of packs smoked/day × number of smoking years. A pack-year is defined as twenty cigarettes smoked every day for one year.

^c^Adjusted for age, BMI, ethnicity, education attainment, alcohol drinking status, passive smoking, family income, and abstinence time.

**Table S7.** Comparison of semen parameters in never smokers and former smokers with different years of quit smoking.

| **Semen parameter^a^** | **Never smoker (n = 1011)** | **Former smoker (n = 84)** | | | | | |
| --- | --- | --- | --- | --- | --- | --- | --- |
|  |  | **< 1 (n = 10)** | | **1-2 (n = 35)** | | **≥ 2 (n = 39)** | |
|  |  | **Unadjusted** | **Multivariable-adjusted^b^** | **Unadjusted** | **Multivariable-adjusted^b^** | **Unadjusted** | **Multivariable-adjusted^b^** |
| **Semen volume (ml)** | Reference | -0.08 (-0.21, 0.06) | -0.01 (-0.13, 0.11) | **-0.10 (-0.18, -0.03)^*^** | -0.07 (-0.14, -0.01) | 0.002 (-0.07, 0.07) | 0.03 (-0.03, 0.09) |
| **Sperm concentration (10^6^/ml)** | Reference | 4.32 (-16.66, 25.26) | 8.57 (-12.50, 29.64) | -11.50 (-22.84, -0.16) | -9.82 (-21.24, 1.59) | -6.70 (-17.47, 4.06) | -6.33 (-17.13, 4.46) |
| **Total sperm count (10^6^)** | Reference | -0.20 (-1.09, 0.68) | 0.21 (-0.62, 1.03) | **-0.66 (-1.14, -0.18)^*^** | -0.47 (-0.92, -0.03) | -0.26 (-0.71, 0.20) | -0.14 (-0.57, 0.28) |
| **Total motility (%)** | Reference | -5.07 (-17.19, 7.05) | -5.56 (-17.64, 6.53) | -2.91 (-9.47, 3.65) | -2.86 (-9.41, 3.69) | 1.64 (-4.59, 7.86) | 2.16 (-4.03, 8.35) |
| **Progressive motility (%)** | Reference | -5.82 (-16.29, 4.64) | -6.54 (-16.93, 3.85) | -2.58 (-8.24, 3.08) | -2.63 (-8.25, 3.00) | 1.91 (-3.46, 7.28) | 2.44 (-2.88, 7.76) |
| **VCL (μm/s)** | Reference | -0.87 (-6.37, 4.63) | -1.82 (-7.30, 3.66) | -0.43 (-3.41, 2.55) | -0.77 (-3.74, 2.20) | 1.27 (-1.56, 4.09) | 1.05 (-1.76, 3.86) |
| **VSL (μm/s)** | Reference | -2.65 (-6.37, 1.08) | -3.38 (-7.08, 0.31) | -0.35 (-2.36, 1.67) | -0.54 (-2.54, 1.46) | 0.19 (-1.72, 2.10) | 0.16 (-1.74, 2.05) |
| **VAP (μm/s)** | Reference | -2.32 (-6.09, 1.46) | -3.10 (-6.85, 0.64) | -0.25 (-2.29, 1.79) | -0.47 (-2.50, 1.56) | 0.21 (-1.73, 2.15) | 0.12 (-1.80, 2.04) |
| **BCF (Hz)** | Reference | 0.43 (0.02, 0.84) | 0.50 (0.09, 0.92) | 0.09 (-0.14, 0.31) | 0.09 (-0.13, 0.32) | -0.02 (-0.23, 0.19) | -0.03 (-0.24, 0.18) |
| **ALH (μm/s)** | Reference | 0.02 (-0.15, 0.19) | 0.02 (-0.15, 0.20) | -0.04 (-0.14, 0.05) | -0.05 (-0.14, 0.05) | 0.06 (-0.03, 0.15) | 0.06 (-0.03, 0.14) |
| **LIN (%)** | Reference | -4.25 (-9.18, 0.67) | -4.72 (-9.69, 0.26) | -0.36 (-3.02, 2.31) | -0.38 (-3.08, 2.31) | -0.91 (-3.43, 1.62) | -0.79 (-3.34, 1.76) |
| **STR (%)** | Reference | -1.74 (-3.64, 0.15) | -1.93 (-3.84, -0.03) | -0.57 (-1.60, 0.45) | -0.63 (-1.66, 0.40) | 0.01 (-0.96, 0.99) | 0.08 (-0.90, 1.05) |
| **WOB (%)** | Reference | -3.51 (-7.71, 0.69) | -3.97 (-8.22, 0.28) | -0.10 (-2.37, 2.17) | -0.17 (-2.47, 2.13) | -1.13 (-3.28, 1.03) | -1.09 (-3.27, 1.09) |
| **MAD (°)** | Reference | 4.22 (-0.60, 9.03) | 4.42 (-0.45, 9.29) | -0.08 (-2.68, 2.53) | -0.09 (-2.73, 2.54) | 1.67 (-0.80, 4.14) | 1.58 (-0.91, 4.08) |

Data are linear regression coefficient (β) and 95%CI. Former smokers with different years of quit smoking versus never smoker: ^*^*P* < 0.05. *P*-values are adjusted by Benjamini-Hochberg with FDR. Bold characters emphasize significant associations.

^a^ALH, amplitude of lateral head displacement; BCF, beat cross frequency; LIN, linearity; MAD, mean angular displacement; STR, straightness; VAP, average path velocity; VCL, curvilinear velocity; VSL, straight line velocity; WOB, curvilinear path wobble.

^b^Adjusted for age, BMI, ethnicity, education attainment, alcohol drinking status, passive smoking, family income, and abstinence time.

**Table S8.** Association between smoking and sperm motility shows a dose-response pattern with increased pack-years.

|  | **Total motility** | | **Progressive motility** | |
| --- | --- | --- | --- | --- |
|  | **Unadjusted** | **Multivariate adjusted^a^** | **Unadjusted** | **Multivariate adjusted^a^** |
| **Constant** | 54.59 | 72.78 | 43.30 | 60.90 |
| **Never smoker** | Reference | Reference | Reference | Reference |
| **Pack-years <5** | 2.20 (-0.05, 4.46) | 1.60 (-0.79, 3.99) | **2.18 (0.22, 4.15)****^*^** | 1.53 (-0.54, 3.61) |
| **Pack-years 5-10** | -0.55 (-3.85, 2.76) | -0.02 (-3.48, 3.44) | -0.69 (-3.57, 2.20) | -0.26 (-3.27, 2.74) |
| **Pack-years ≥10** | 2.87 (-1.47, 7.21) | **6.02 (1.41, 10.63)****^*^** | 2.35 (-1.43, 6.13) | **5.52 (1.52, 9.53)^**^** |
| **Trend test p value^b^** | 0.317 | **0.032** | 0.382 | **0.028** |

Data are linear regression coefficient (β) and 95%CI. Bold characters emphasize significant associations.

^a^Adjusted for age, BMI, ethnicity, education, drinking status, family income, passive smoking, abstinence period.

^b^The *P*-value for trend test across pack-year categories with the never smoker group as the reference category.

**Table S9.** Coefficients from linear regression for pack-years in relation to semen volume by categories of age, BMI, income, passive smoking and alcohol drinking.

| **Subgroups** | **Never smoker** | **Ever smoker (≥ 10 pack-years)** | |  |
| --- | --- | --- | --- | --- |
|  |  | **β (95% CI)** | ***P*** | ***P* for interaction** |
| **Age, years** | | | |  |
| < 35 (n = 1349) | Reference | -0.12 (-0.19, -0.06) | < 0.001 | 0.117 |
| ≥ 35 (n = 282) | Reference | -0.04 (-0.12, 0.04) | 0.334 |  |
| **BMI, kg/m^2^** | | | |  |
| < 24 (n = 707) | Reference | -0.07 (-0.14, 0.01) | 0.083 | 0.433 |
| ≥ 24 (n = 924) | Reference | -0.11 (-0.18, -0.05) | < 0.001 |  |
| **Family income** | | | |  |
| < 100,000 yuan (n = 596) | Reference | -0.17 (-0.25, -0.08) | < 0.001 | 0.011 |
| ≥ 100,000 yuan (n = 1035) | Reference | -0.03 (-0.08, 0.03) | 0.348 |  |
| **Passive smoking** | | | |  |
| No (n = 872) | Reference | -0.06 (-0.13, 0.02) | 0.156 | 0.557 |
| Yes (n = 759) | Reference | -0.09 (-0.16, -0.03) | 0.007 |  |
| **Alcohol drinking** | | | |  |
| Never (n = 723) | Reference | -0.17 (-0.26, -0.08) | < 0.001 | 0.022 |
| Ever (n = 908) | Reference | -0.05 (-0.11, -0.001) | 0.046 |  |

Data are linear regression coefficient (β) and 95%CI.

**Table S10.** Coefficients from linear regression for pack-years in relation to total sperm count by categories of age, BMI, income, passive smoking and alcohol drinking.

| **Subgroups** | **Never smoker** | **Smoker (≥ 10 pack-years)** | |  |
| --- | --- | --- | --- | --- |
|  |  | **β (95% CI)** | ***P*** | ***P* for interaction** |
| **Age, years** | | | |  |
| < 35 (n = 1349) | Reference | -0.43 (-0.88, 0.01) | 0.054 | 0.814 |
| ≥ 35 (n = 282) | Reference | -0.35 (-0.85, 0.15) | 0.169 |  |
| BMI, kg/m^2^ | | | |  |
| < 24 (n = 707) | Reference | -0.48 (-1.01, 0.05) | 0.079 | 0.599 |
| ≥ 24 (n = 924) | Reference | -0.30 (-0.71, 0.11) | 0.155 |  |
| Family income | | | |  |
| < 100,000 yuan (n = 596) | Reference | -0.70 (-1.22, -0.17) | 0.009 | 0.079 |
| ≥ 100,000 yuan (n = 1035) | Reference | -0.10 (-0.50, 0.31) | 0.640 |  |
| Passive smoking | | | |  |
| No (n = 872) | Reference | 0.03 (-0.51, 0.57) | 0.919 | 0.069 |
| Yes (n = 759) | Reference | -0.61 (-1.04, -0.18) | 0.005 |  |
| Alcohol drinking | | | |  |
| Never (n = 723) | Reference | -0.41 (-1.00, 0.18) | 0.170 | 0.802 |
| Ever (n = 908) | Reference | -0.32 (-0.69, 0.06) | 0.096 |  |

Data are linear regression coefficient (β) and 95%CI.
